# Variability in a dominant block to SIV early reverse transcription in rhesus monkey cells predicts *in vivo *viral replication and time to death

**DOI:** 10.1186/1743-422X-7-79

**Published:** 2010-04-26

**Authors:** Thomas F Rogers, So-Yon Lim, TJ Sundsvold, Tiffany Chan, Ariel Hsu, Norman L Letvin

**Affiliations:** 1 Division of Viral Pathogenesis, Beth Israel Deaconess Medical Center, Harvard Medical School, Boston, Massachusetts 02115, USA

## Abstract

While it has long been appreciated that there is considerable variability in host containment of HIV/SIV replication, the determinants of that variability are not fully understood. Previous studies demonstrated that the degree of permissivity of a macaque's peripheral blood mononuclear cells (PBMC) for infection with simian immunodeficiency virus (SIV) *in vitro *predicted that animal's peak plasma virus RNA levels following SIV infection *in vivo*. The present study was conducted to define the mechanisms underlying the variable intrinsic susceptibility of rhesus monkey PBMC to SIVsmE660 infection. In a cohort of 15 unrelated Indian-origin rhesus monkeys, infectability of PBMC of individual animals with SIVsmE660, as defined by tissue culture infectious dose (TCID_50_), varied by more than 3 logs and was a stable phenotype over time. Susceptibility of a monkey's PBMC to wild type SIVsmE660 infection correlated with the susceptibility of that monkey's PBMC to infection with VSV-G pseudotyped SIVsm543-GFP. Moreover, the permissivity of an individual monkey's PBMC for infection with this construct correlated with the permissivity of a B-lymphoblastoid cell line (B-LCL) generated from PBMC of the same animal. We found that the degree of intrinsic resistance of monkey B-LCL correlated with the copy number of early reverse transcription (ERT) SIV DNA. The resistance of monkey B-LCL to SIVsmE660 replication could be abrogated by preincubation of cells with the SIV virus-like particles (VLPs) and SIV resistance phenotype could be transferred to a SIV susceptible B-LCL through cell fusion. Finally, we observed a positive correlation between susceptibility of monkey B-LCL to SIV infection with a VSV-G pseudotyped SIV-GFP construct *in vitro *and both the peak plasma virus RNA levels *in vivo *and time to death following wild type SIV infection. These findings suggest that a dominant early RT restricting factor that can be saturated by SIV capsid may contribute to the variable resistance to SIV infection in rhesus monkey B-LCL and that this differential intrinsic susceptibility contributes to the clinical outcome of an SIV infection.

## Introduction

Humans vary in their susceptibility to human immunodeficiency virus type 1 (HIV-1) acquisition and in the level of HIV-1 replication following infection [1,2]. Virus phenotype, the magnitude of cytotoxic T lymphocyte response, major histocompatability complex (MHC) class I haplotype, and chemokine receptor polymorphisms have all been shown to contribute to this variability [3-5]. Recent studies suggest that further undefined host factors are also contributing to the level of virus control in the HIV-1-infected individual [6].

Some of the undefined host factors contributing to HIV-1 containment may be responsible for the variable ability of cells to be infected by and sustain replication of these viruses. It has been shown that permissiveness for HIV-1 varies substantially between isolated primary cells Permissiveness, the ability of cells to be infected and sustain the replication of HIV-1, also varies substantially between isolated primary cells of individuals [7]. It has been shown that the permissiveness of isolated primary rhesus monkey lymphocytes for SIV infection correlates with *in vivo *viral set point [8].

The variability in permissiveness observed in rhesus monkey PBMC for SIV replication can be shaped by dominant and nondominant mechanisms: through the altered expression of required host factors and/or virus restricting molecules [9]. The ability of a cell to become infected by HIV/SIV requires cellular expression of diverse proteins and HIV/SIV replication can be repressed by the cellular expression of a number of restriction factors including TRIM5α and APOBEC3G [10,11]. While the restriction factor TRIM5α appears to be under positive selection and is highly polymorphic in a given species, there is little evidence that this genetic variability has functional consequences [12-15].

We set out to identify genetic mechanisms underlying the variable susceptibility to lentivirus replication in a primate species through an analysis of the differential rhesus monkey permissivity of lymphocytes for SIV replication. The SIVsmE660-infected rhesus monkey provides a powerful model system for elucidating the genetic contribution to virus control. Studies can be done with a single, defined challenge stock of virus, and monkeys can be selected for evaluation with specified genetic characteristics. There is a well-documented variability in both peak and set point replication of this virus in genetically disparate rhesus monkeys. Moreover, there is a clear correlation between the *in vitro *susceptibility of PBMC of rhesus monkeys to infection (TCID_50_) with this virus isolate and the extent of early virus replication in these animals following experimental infection [8]. In the present study, we explored the mechanisms resulting in the variation of rhesus monkey PBMC permissiveness for SIVsmE660 replication and examined the contribution of this process to the pathogenicity of the virus *in vivo*.

## Methods

### *In vitro *susceptibility assay

PBMC were separated by centrifugation through Ficoll and CD8+ cells were depleted from PBMC cultures using CD8 Dynabeads (Dynal). CD8+- depleted PBMC were resuspended in RPMI 1640 with 6.25 ug of ConA per ml, 10% fetal calf serum (FCS), and 10% interleukin-1 (IL-2) at a density of 2 × 10^6 ^PBMC per ml. Three days after initial culture, aliquots of 40,000 PBMC were incubated for 4 hours with 150 ul of serial 10-fold dilutions of a cell-free virus stock of SIVsmE660. PBMC were washed with Hanks balanced salt solution to remove residual virus, resuspended in RPMI 1640 with 10% FCS and 10% IL-2, and cultured in 96-well plates. Cultural supernatant were collected at days 0,3,7,10, and 13 after infection and monitored for the presence of virus by antigen capture assay for SIV p27 antigen (Coulter Corp.) The minimal TCID or endpoint titer of virus required to infect the cells of each donor was determined as the last dilution in which virus was detected by 13 days after infection.

### Viruses and cell lines

The majority of studies were conducted using SIVsmE660[16]. The molecular clone of SIVsmE660, SIVsmE543-3 was used to generate VSV-G pseudotyped SIVsm543-GFP. The SIVsmE543 vector was created by inserting SalI-NotI fragment of pSIV.GFP into the env gene of SIVsmE543-3. VSV-G pseudotyped SIVsm543-GFP, HIV-GFP, Murine Leukemia Virus (MLV-GFP), and SIVmac-GFP were produced as previously described [17]. SIV virus like particles (VLP) were generated by insertion of a stop codon into the ORF of SIV reverse transcriptase (RT). B-lymphoblastoid cell lines (B-LCL) were generated by incubating rhesus monkey PBMC with 100 ul herpes papio, washed and seeded into 96-well plates in RPMI 1640 with 20% FCS. PBMC were fed twice weekly with 50% medium changes and transferred to T25 flasks after 4 weeks.

### Real-time RT-PCR quantification

The quantification of early reverse transcription, late reverse transcription, and integrated SIV DNA copy number was modified from a previously described method [18]. At 2, 3, 6, 12, 24, and 48 hours following infection of B-LCL with VSV-G pseudotyped SIVsm543-GFP, cells were collected and DNA was isolated with DNAeasy Tissue kit (Qiagen). The primer sets used to detect each sequence were as follows: early RT forward, 5'-AAGCAAGTGTGTGTTCCCATCT-3'; early RT reverse, 5'-CCTCGGTTTCCCAAAGCAGAA-3'; late RT forward, 5'-AAGCAAGTGTGTGTTCCCATCT-3'; late RT reverse, 5'-CACTTACCTGCAACCGGAGG-3'; integrated forward, 5'-GCTGCCGATTGGGATTTACAAC-3'; integrated reverse, 5'-AATGTCTGATCCTCTTGGCTCTC-3'. Quantification was performed with an Applied Biosystems 7300 real-time PCR system (Foster City, CA). GAPDH control primers and probe was purchased through Applied Biosystems.

### Fusion assay

B-LCL were stained with 1 ul of Oregon Green or 2 ul of Vybrant DiD (Invitrogen) for 1 hour. Cells were washed with RPMI 1640 and allowed to rest for 30 minutes. B-LCL populations were mixed together and added to 2 ml of 50% PEG for 1 minute. B-LCL were washed and allow to rest in RPMI 1640 with 10% FCS for twelve hours. Double positive fusion events were sorted using flow cytometry and infected with VSV-G pseudotyped SIVsm543-GFP.

## Results

### Variation in rhesus monkey PBMC susceptibility to SIV replication

**Figure 1:**
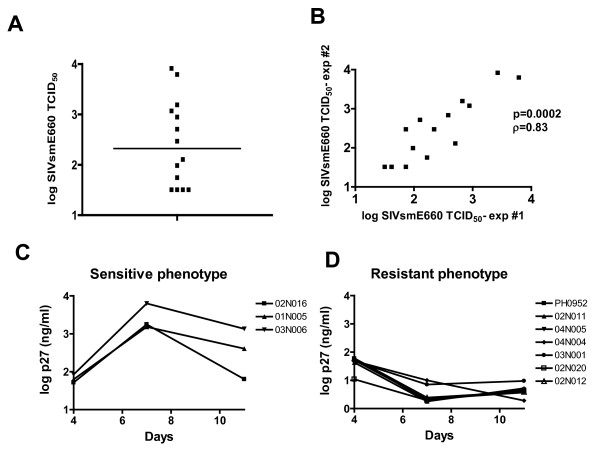
**Susceptibility of PBMC of 14 rhesus monkeys to in vitro SIVsmE660 infection**. (A) CD8+ T lymphocyte-depleted PBMC were infected with SIVsmE660 and p27 production was assayed in the culture supernatant by ELISA. Susceptibility to in vitro infection is expressed as the relative TCID_50 _of the SIVsmE660 virus stock using the Spearman-Karber method. (B) PBMC of the same animals were assayed again for in vitro susceptibility to SIVsmE660 after a three month interval. A significant positive correlation was observed between the values obtained from PBMC of each monkey in these repeated assays. (C, D) CD8+ T lymphocyte-depleted PBMC were infected with SIVsmE660, samples collected serially and grouped for display as (C) sensitive or (D) resistant to SIV replication on the basis of their TCID50 values.

We established a cohort of 15 unrelated Indian-origin rhesus monkeys to investigate the impact of cellular SIV permissivity on *in vivo *viral replication. CD8+ T lymphocyte-depleted PBMC from each monkey were evaluated for their relative ability to sustain SIVsmE660 replication *in vitro*. Lectin-stimulated CD8+ lymphocyte-depleted PBMC were exposed to serial dilutions of SIVsmE660. Replication of virus was assessed by measuring SIV p27 antigen in culture supernatants and a minimal tissue culture infectious dose (TCID_50_) of virus was defined for each population of PBMC. We observed a broad range of permissiveness of these PBMC for SIVsmE660 replication, with rhesus PBMC populations differing by as much as 4 logs of the virus needed to initiate infection (Fig. 1A).

To determine whether this PBMC permissiveness for SIV replication is a stable phenotype, the TCID_50 _assay was repeated after a 3 month interval on CD8+ T lymphocyte-depleted PBMC of the same 15 monkeys. The relative susceptibility of these PBMC populations was remarkably similar, with a correlation of data demonstrating a Spearman p = 0.0002 and ρ = 0.8345 (Fig. 1B).

To assess the nature of this variable susceptibility phenotype in these PBMC, we examined the kinetics of viral replication in each PBMC population by sequential analysis of p27 production over time. We observed very different kinetics of SIV replication in these rhesus monkey PBMC, with susceptible cell lines supporting robust viral replication and resistant cells supporting little to no viral replication (Fig 1C and 1D). Rhesus monkey PBMC that exhibited low TCID50 values did not have delayed replication kinetics; rather, they showed a persistent resistance to viral replication.

### Rhesus monkey PBMC susceptibility to SIV replication is independent of entry

Since genetic polymorphisms have been described that impact HIV-1 replication through modulation of viral entry, we sought to determine whether this variable susceptibility to SIV replication was a consequence of differential SIV entry or represented a post-entry phenomenon. To explore these issues, we created a VSV-G-pseudotyped SIVsmE660 construct that expressed GFP. The molecular clone of SIVsmE660, SIVsmE543, was engineered to produce VSV-G pseudotyped SIVsme543 through the deletion of the SIV *env *gene and complementation with a VSV-G-encoding vector. Furthermore, the SIVsme543 plasmid was manipulated by insertion of GFP ORF into the *env *gene of SIV543 (SIVsm543-GFP) to allow the expression of GFP following SIV integration, transcription and translation of viral proteins. This construct allowed a simple and rapid quantification of SIV infected cells by flow cytometric analysis. Moreover, since the VSV-G protein mediated viral entry into a broad range of cell types, variability in infection by this construct must reflect a gp120-CD4 independent phenomenon.

To determine if rhesus PBMC susceptibility to wild type SIVE660 is associated with susceptibility of these cells to a single-cycle pseudotyped SIV construct, rhesus monkey PBMC were infected with the VSV-G pseudotyped SIVSM543-GFP and analyzed by flow cytometry for % GFP-positive cells (Figure 3A). Rhesus monkey PBMC susceptibility to wild type SIVsmE660 virus was defined by TCID_50_, while susceptibility to the single cycle SIVsm543-GFP construct was defined as the % GFP+ PBMC 48 hrs post-infection. Rhesus monkey PBMC exhibited varied susceptibility to the single cycle VSV-G pseudotyped SIVSsm543-GFP, and a significant correlation was observed for each cell population between % SIVsm543-GFP+ cells and the TCID_50 _of these cells for wild type SIVsmE660 (Figure 3A). These data suggest that the differential permissivity of rhesus monkey PBMC to SIV replication is entry independent. Additionally, because the permissivity phenotype was manifested by the expression of GFP following a single cycle of replication, the relative blockage of viral replication must occur in the virus life cycle between early reverse transcription and post-integration viral expression. Moreover, the expression of this phenotype does not require multiple rounds of viral replication. Only one monkey's PBMC demonstrate a disparity between their permissivity to wild type virus and VSV-G pseudotyped SIVsm543-GFP.

### Rhesus monkey PBMC susceptibility to SIV infection correlates with B-lymphoblastoid cell line susceptibility to SIV infection

The susceptibility of rhesus monkey CD4+ T lymphocytes to SIV infection is dependent on the activation state of the T cells, the replication rate of the cells, as well as the expression level of CCR5, which reflects the memory phenotype of these cells (7,8,9). Additionally, the relative representation of CD4+ T cells in a PBMC population could impact the quantitation of the relative susceptibility of PBMC to SIV infection. To rule out a contribution of these well-defined factors to the variability of rhesus monkey lymphocyte susceptibility to SIV replication, we assessed the permissivity of rhesus monkey B-lymphoblastoid cell lines (B-LCL) to VSV-G pseudotyped SIVsm543-GFP infection. In addition to facilitating rapid screening of large numbers of cell populations, analysis of B-LCL would establish if this differential permissivity for SIV replication is T cell-specific or is manifested in other cell types.

**Figure 2:**
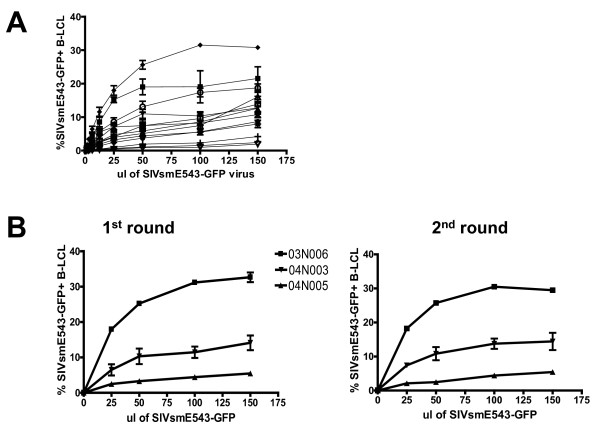
**Rhesus monkey B-LCL exhibit differential susceptibility to SIVsmE660 infection**. (A) B-LCL were generated from PBMC of 15 rhesus monkeys and infected with varying dilutions of VSV-G pseudotyped SIVsmE543-GFP. SIVsmE543-GFP susceptibility was defined by flow cytometry as % GFP+ B-LCL following infection (B) B-LCL were infected with SIVsmE660-GFP and sorted for the GFP negative cell population. These GFP negative cells were reinfected with SIVsmE543-GFP and, again, analyzed by flow cytometry.

We generated B-LCL from 15 unrelated Indian-origin rhesus monkeys and analyzed B-LCL and PBMC from the same animals for susceptibility to infection by VSV-G pseudotyped SIVsm543-GFP. Following infection of rhesus monkey B-LCL with this construct, we observed a broad range in B-LCL susceptibility to SIV infection, varying from 1% to 30% of target cells infected (Fig. 2A). To determine whether B-LCL permissivity for SIV replication is a stable phenotype, B-LCL from the same animals were infected a second time. There was a significant correlation between susceptibility phenotypes for experiment #1 and #2 for all 15 animals (data not shown). Additionally, B-LCL were generated from the same monkeys two months later and assayed for susceptibility using the VSV-G pseudotyped SIVsm543-GFP construct. There was a significant correlation between the relative susceptibility to SIV infection of the two populations of B-LCL for all animals (data not shown).

The differential susceptibility of B-LCL populations from different monkeys for SIV replication could be a consequence of two possible mechanisms: a subpopulation of variable size in a B-LCL population could be permissive or the total B-LCL population could be uniform in its relative resistance or susceptibility to SIV infection. To examine these two possibilities, B-LCL were infected with the VSV-G pseudotyped SIVsm543-GFP, GFP negative cells were sorted, and the GFP negative cells were reinfected with the same viral construct. No significant change in relative susceptibility to SIV infection was observed following the second round of infection (Fig. 2B). This observation suggests that the total population of B-LCL from each animal exhibits a uniform susceptible or resistant phenotype.

**Figure 3:**
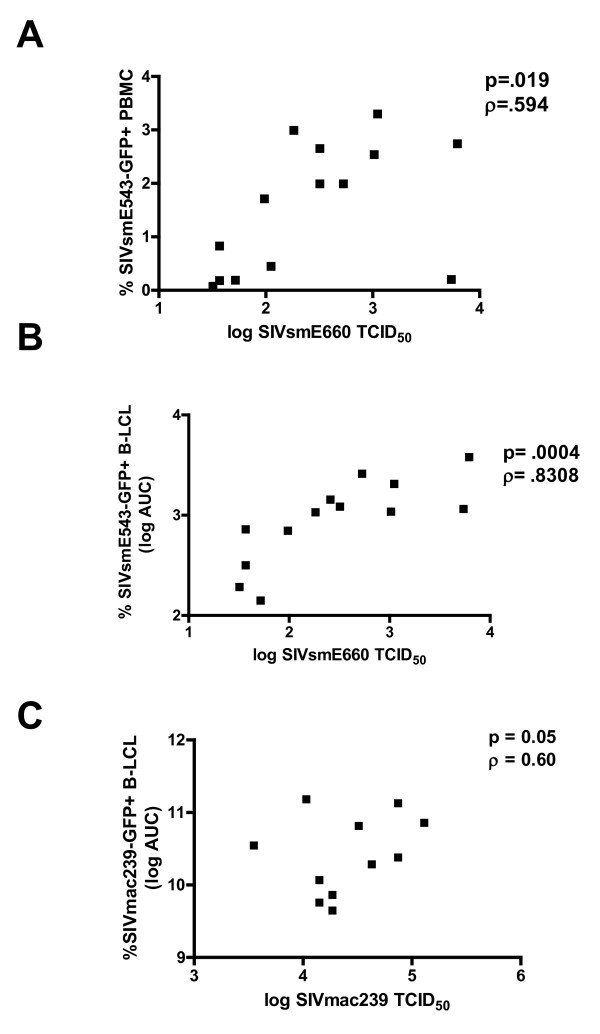
**Positive correlation between rhesus monkey PBMC susceptibility to infection with wild type SIVsmE660 and a single cycle VSV-G pseudotyped SIVsmE660-GFP construct**. (A) PBMC susceptibility to SIVsmE660 replication was defined by TCID_50_, and SIVsmE543-GFP infection was defined as % GFP+ PBMC following infection with VSV-G pseudotyped SIVsmE660-GFP determined using flow cytometric analysis. (B) Positive correlation between rhesus monkey PBMC susceptibility to SIVsmE660 infection, as defined by TCID_50_, and area under the curve (AUC) of % GFP+ B-LCL following infection with serial dilutions of VSV-G pseudotyped SIVsmE543-GFP. (C) Positive correlation between rhesus monkey PBMC susceptibility to SIVmac239 infection, as defined by TCID_50_, and area under the curve (AUC) of % GFP+ B-LCL following infection with serial dilutions of VSV-G pseudotyped SIVmac239-GFP.

To determine the relationship between the susceptibility of a given population of B-LCL to VSV-G pseudotyped SIVsm543-GFP infection and the susceptibility of PBMC from the same monkey to wild type virus replication, we assayed these cell populations from a cohort of rhesus monkeys. In these experiments, we defined PBMC susceptibility to wild type SIVsmE660 by TCID_50_. To represent SIV permissivity of each cell line accurately over a range of multiplicities of infection (MOIs), B-LCL susceptibility was defined as the area under the curve (AUC) of % GFP+ cells over 4 serial viral dilutions following infection with the single cycle VSV-G pseudotyped SIVsm543-GFP. We observed a clear positive correlation between PBMC susceptibility to wild type SIVsmE660 infection and the permissivity of B-LCL from the same monkey to single cycle VSV-G pseudotyped SIVsm543-GFP (Spearman p value = 0.0004, ρ = 0.83(Fig. 3B). Similarly, there was a significant correlation between permissivity of PBMC to infection with wild type SIVmac239 and B-LCL to infection with VSV-G pseudotyped SIVmac239-GFP (Fig. 3C). These data indicate that differential intraspecies permissivity for SIV replication in rhesus monkey lymphocytes is not dependent on the activation or memory state of CD4+ T cells, is not entry dependent, does not reflect the susceptibility of a subpopulation of cells, is not dependent on multiple rounds of infection, is not specific for SIVsmE660 and is manifested at a stage of viral replication prior to viral gene expression.

### SIV resistance in rhesus monkey cells correlates with resistance to primate immunodeficiency viruses, but not to other viruses

**Figure 4:**
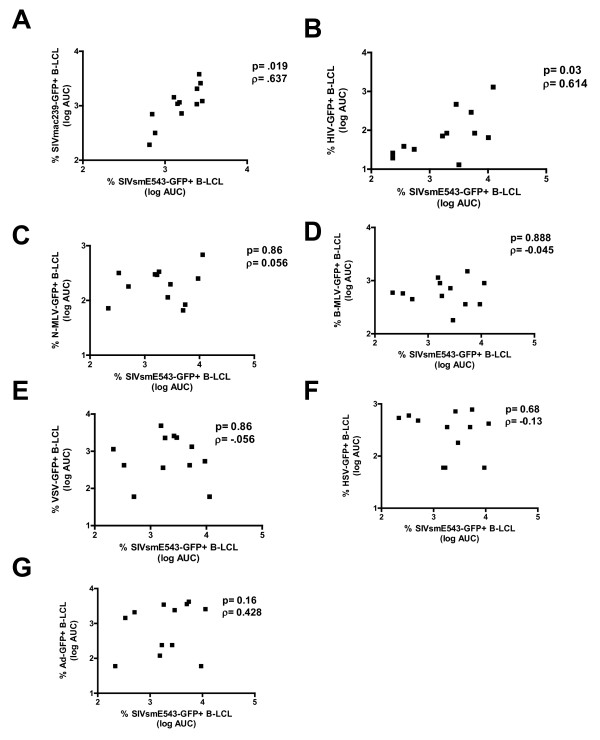
**Rhesus monkey B-LCL susceptibility to SIVsmE543 infection correlates with susceptibility to SIVmac239 and HIV-1 infection**. (A) Positive correlation between B-LCL susceptibility to VSV-G pseudotyped SIVsmE543-GFP infection and VSV-G pseudotyped SIVmac239-GFP infection. (B) Positive correlation between B-LCL susceptibility to VSV-G pseudotyped SIVsmE543-GFP infection and VSV-G pseudotyped HIV-GFP infection. No positive correlation with susceptibility to VSV-G psuedotyped SIVsmE543-GFP was observed when rhesus monkey B-LCLs were infected with serial dilutions of N-MLV-GFP (C), B-MLV-GFP (D), Ad-GFP (E), VSV-GFP (F), or HSV-GFP (G).

To determine the range of viruses whose replication is associated with SIV replication in rhesus monkey B-LCL, we infected 15 B-LCL populations with VSV-G pseudotyped SIVmac239-GFP, HIV-GFP, N-MLV-GFP, and B-MLV-GFP at varying dilutions. B-LCL infected with SIVmac239-GFP and SIVsm543-GFP exhibited differential inflexibility which was significantly correlated (Fig. 4A). Furthermore, we found that HIV-GFP susceptibility correlated with SIVsm543-GFP susceptibility (Fig. 4B). However, rhesus monkey B-LCL permissivity for adenovirus-GFP, VSV-GFP, HSV-GFP, N-MLV-GFP and B-MLV-GFP did not correlate with permissivity for SIV or HIV (Fig 4C, D, E, F, G). This study suggests that the relative block to SIVsmE660 infection in B-LCL acts through a mechanism that can also restrict other SIVs and HIV, but allows the replication of other viruses.

### Rhesus monkey cells exhibit a variable block to early reverse transcription of SIV

**Figure 5:**
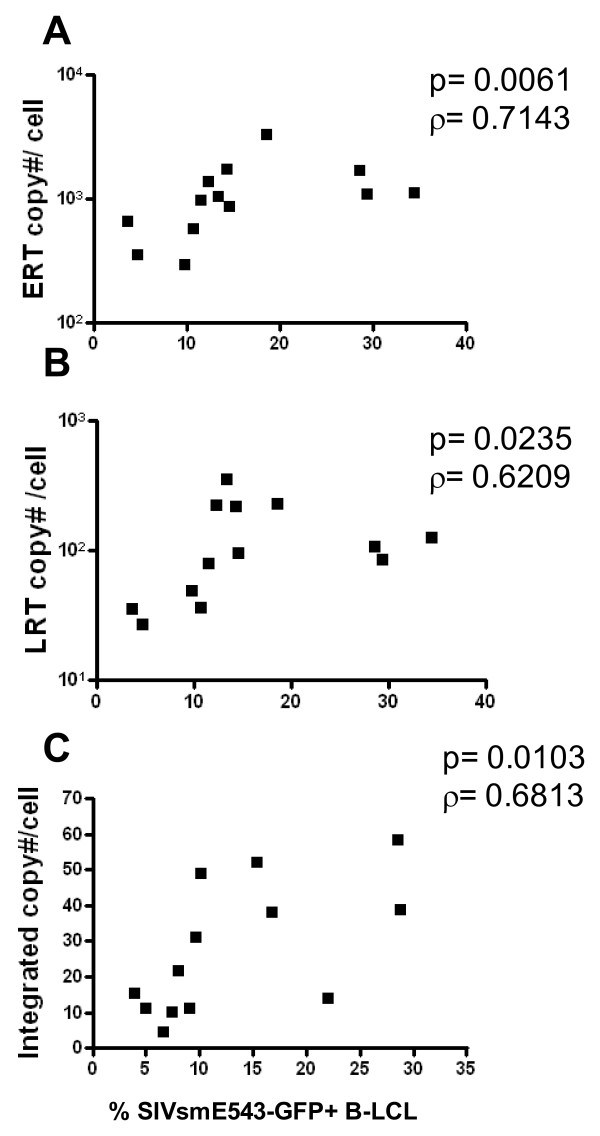
**Rhesus monkey cells exhibit a variable block to early reverse transcription of SIV**. Rhesus monkey B-LCLs were infected with serial dilutions of VSV-G pseudotyped SIVsmE543-GFP and DNA was collected a 5 time points. The quantity of early reverse transcription, late reverse transcription, and integrated viral DNA was assessed by real time PCR.

We then sought to determine whether intraspecies host genetic variability can influence HIV/SIV replication at steps in the viral life cycle other than viral entry. Fifteen B-LCL with variable resistance to SIV replication were infected to assess the contributions of specific intracellular blocks to SIV replication on the resistance phenotype of these cells. By assaying the progression of VSV-G pseudotyped SIVsmE660 through its life cycle, we evaluated key replication stages that might correlate with host SIV permissiveness. DNA was collected at 5 time points following infection for quantitation of early reverse transcription (early RT), late reverse transcription (late RT), and integrated viral DNA copy number using quantitative real time PCR. Spearman correlations were performed to evaluate the relationship between copy number/cell of each viral DNA species and the SIVsmE660 permissivity of that cell. A significant positive correlation was observed between copy number of early RT, late RT, and integrated copy number and SIV permissivity (Fig. 5). These data suggest that the relative resistance to SIV infection is manifest before early reverse transcription, but after viral entry. Additionally, they suggest that there is a wide differential in the ability of different rhesus monkey cell populations to mediate an early reverse transcription blockade.

### Rhesus monkey cells can block early RT of SIV replication through a dominant, saturable restriction factor

**Figure 6:**
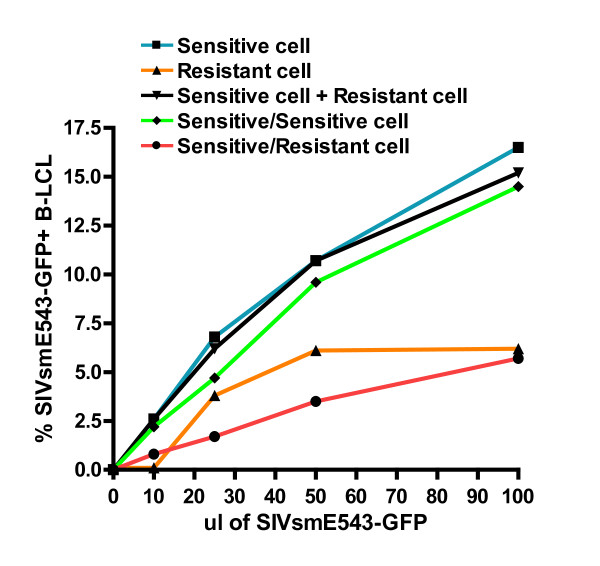
**Fusion of SIV resistant and susceptible rhesus monkey B-LCLs demonstrates a dominant SIV resistance phenotype**. SIV susceptible and resistant B-LCLs were labeled with either Vybrant DID (Invitrogen) or Oregon Green (Invitrogen). Susceptible and resistant cell lines were fused using PEG incubation and double positive fusion events were sorted by flow cytometry. Fused cells were subsequently infected with SIVsmE543-GFP and quantified for % GFP+ by flow cytometry.

On the basis of these data, we hypothesized that rhesus monkey B-LCL express a protein or variable forms of a protein that inhibit early reverse transcription by interacting with incoming lentiviral capsid. To test this hypothesis, we first examined the dominance of the SIV resistance phenotype in B-LCL. To determine if the resistance of B-LCL derived from some rhesus monkeys to SIV replication is a dominant or non-dominant phenotype, we performed SIV infection assays on cells that were fusions of SIV-resistant and SIV-sensitive B-LCL. By examining the SIV susceptibility phenotype in a cell that is a fusion between a resistant and a susceptible B-LCL, we could determine if resistance to SIV replication in B-LCL is mediated by a factor that is present in resistant cells or reflects the absence of a factor necessary for early reverse transcription. Since nonfused cells and homokaryotypic fusions would impact the MOI of the VSV-G pseudotyped SIVsm543-GFP infection, we generated PEG-mediated fusions of resistant and susceptible cell lines that were stained with intracellular dyes. Heterokaryotypic fusion products could then be isolated through detect of the intermixing of the dyes by flow cytometry. Using this strategy, we showed that a sensitive/sensitive cell fusion maintained a sensitive phenotype, while a resistant/sensitive cell fusion was resistant to SIV infection (Fig. 6). These data suggest that a dominant factor exists in resistant B-LCL that can inhibit SIV replication.

**Figure 7:**
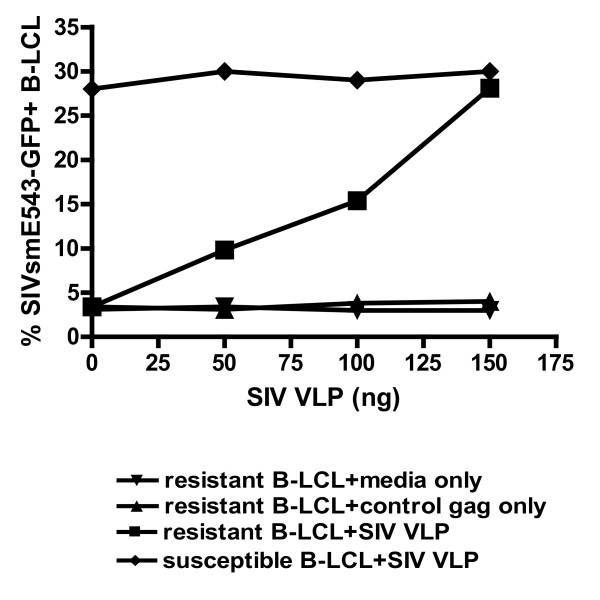
**Enhancement of susceptibility of B-LCL to SIV infection following incubation with SIV virus-like particles**. Rhesus monkey B-LCLs were incubated with media only, uncleaved Gag protein or SIV virus like particles (VLPs) for four hours followed by infection with VSV-G pseudotyped SIVsmE543-GFP. Preincubation of B-LCL with VLPs augmented the % GFP+ B-LCL following SIVsmE543-GFP infection.

To determine if a protein or variable forms of a protein inhibit early reverse transcription by interacting with incoming lentiviral capsid, we examined the impact of virus-like particles on SIV resistance in B-LCL populations. The strategy we adopted for this study was to preincubate resistant B-LCL with nonreplicating virus-like particles (VLPs) which contain intact viral capsid. In doing so we should saturate a capsid binding factor and reverse the dominant block of early reverse transcription and SIV replication. We engineered an SIVsmE660 construct with a deletion in reverse transcriptase and produced VLPs through transfection of the plasmid into 293T cells. We preincubated rhesus monkey B-LCL with SIVsmE660 VLPs and then infected the cells with SIVsm543-GFP (Fig. 7). We observed an increase in SIVsmE660 replication in resistant cell lines as we added increasing quantities of VLPs. In fact, the addition of 150 ng of VLPs led to the complete loss of resistance to SIV replication in the cell line. These findings suggest that a dominant early RT restricting factor that can be saturated by SIV capsid may contribute to differential resistance to SIV infection in rhesus monkey B-LCL.

### Rhesus monkey B-LCL susceptibility to SIV-GFP correlates with in vivo viral load and time to death following infection of rhesus monkeys with SIV

**Figure 8:**
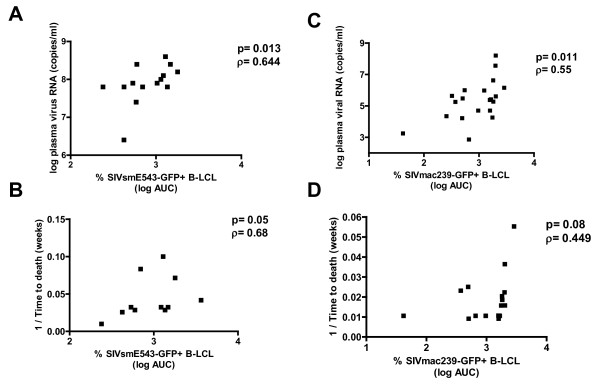
**B-LCL susceptibility to VSV-G pseudotyped SIV-GFP correlates with in vivo plasma viremia and time to death following rhesus monkey challenge with SIVmac251 or SHIV-89.6P**. Positive correlation between % GFP+ B-LCL following VSV-G pseudotyped SIVsmE543-GFP in vitro infection of rhesus monkey B-LCL and (A) in vivo peak plasma viremia of monkeys (day 14 post-infection) as measured by RT assay, and (B) time to death following in vivo infection with SHIV-89.6P. Positive correlation between % GFP+ B-LCL following VSV-G pseudotyped SIVmac239-GFP in vitro infection of rhesus monkey B-LCL and (C) in vivo peak plasma virus RNA levels of monkeys (day 14 post-infection) and positive trend with (D) time to death following in vivo infection with SIVmac251.

To determine if this variable intracellular blockade of primate immunodeficiency virus replication impacts *in vivo *viral replication and clinical outcome in SIV-infected rhesus monkeys, we evaluated the permissivity of B-LCL generated from 14 monkeys for VSV-G pseudotyped SIVsm543-GFP infection. Then, following infection of the monkeys with SHIV89.6P, we assessed the correlation between the *in vitro *permissiveness of these B-LCL for replication of this virus and the *in vivo *peak plasma virus RNA levels and time to death following infection of rhesus monkeys with wild type virus (Fig. 8A, B). Additionally, we generated B-LCL from prechallenge PBMC of another cohort of rhesus monkeys that were infected with wild type SIVmac251 and assessed these B-LCL for susceptibility to SIVmac239-GFP replication. We observed a significant positive correlation and positive trend between *in vitro *B-LCL susceptibility to infection by these VSV-G pseudotyped SIV constructs and both peak viremia and time to death in the wild type SIV-infected monkeys, respectively (Fig. 8C, D). Monkeys whose B-LCL demonstrated a relative block to early RT exhibited a lower set point viral load following challenge *in vivo *with SIVmac251. These data indicate that *in vitro *permissivity of B-LCL for SIV replication is a reliable predictor of *in vivo *viral replication and clinical outcome.

## Discussion

The variability in the clinical course of HIV-infected humans and SIV-infected monkeys is likely a consequence of both host and viral factors. The present study was initiated to begin an exploration of the impact of the intrinsic immune response to lentiviral infection on clinical course in rhesus monkeys. We attempted to determine the importance of CD4+ T cell permissiveness for SIV replication on clinical outcome in monkeys and define a mechanism for the variability of this permissivity. The present experiments built upon earlier studies of this phenomenon which showed that *in vitro *CD4+ T cell susceptibility to SIV infection is correlated with *in vivo *peak virus load [8]. We observed that differential susceptibility to SIV replication is a stable phenotype and was reproducible with a single cycle VSV-G pseudotyped viral construct. Moreover, B-LCL generated from a PBMC population exhibited a relative permissiveness for SIV replication that is similar to the relative permissiveness of those PBMC. We also demonstrated a significant correlation between the susceptibility of B-LCL to a VSV-G pseudotyped SIVmac construct and the *in vivo *virus set point and time to death in rhesus monkeys infected with SIVmac251. The fact that B-LCL susceptibility can predict the variability observed in PBMC permissiveness for lentivirus replication indicates that the intracellular control of retrovirus replication is of central importance in determining the infectability of CD4+ T cells and the clinical outcome of SIV infections. This positive correlation between PBMC and B-LCL permissiveness for SIV replication also indicates that the variability in virus replication between rhesus monkeys results from a post-entry block of SIV replication that is manifested in diverse lymphocyte populations.

The infection of rhesus monkeys with SIV is a powerful model for HIV infection in humans and is of critical importance for drug and vaccine development. The variability in the level of virus replication in monkeys following SIV infection necessitates the use of large numbers of animals to appropriately power vaccine trials. Prescreening monkeys' B-LCL for susceptibility to the replication of VSV-G-pseudotyped SIV-GFP should allow a prediction of the relative susceptibility of monkeys to SIV replication following *in vivo *virus challenge. This should facilitate the preselection of animals for a study with similar permissivities, improving the power of the study and clarifying the impact of the evaluated intervention.

Following the identification of this phenotype of variable permissiveness for SIV replication in lymphocytes of rhesus monkeys, we conducted a series of studies to begin defining the mechanism underlying this observed intraspecies variability. We established that the relative block in SIV replication was not dependent on multiple cycles of SIV replication and was due to a differential ability of monkey lymphoctyes to block early reverse transcription of SIV. Moreover, we demonstrated that the relative block in early RT was a dominant phenotype. This dominant block to SIV replication could be transferred to a highly susceptible monkey B-LCL population by cell fusion. Additionally, the block to early RT could be overcome by preincubation of SIV resistant cell lines with virus-like particles. These data suggest that the block to early RT may involve the binding of capsid of incoming virions.

Several gene products have been implicated to date in the control of HIV/SIV entry and cellular immunity, including CCR5, MHC, and KIR. The present study demonstrates another mechanism that contributes to SIV control in monkeys. Several intrinsic anti-viral immune molecules have been shown to inhibit retroviral replication by preventing early reverse transcription *in vitro*. APOBEC-3G, a cellular cytidine deaminase, induces C to U mutations in the negative strand of the HIV DNA, which results in a reduced number of infectious HIV progeny virions[11]. Products of several members of the *trim *gene family have the capacity to inhibit virus replication. Transfection of rhesus monkey TRIM5α into feline fibroblast cells potently blocks early reverse transcription of HIV-1, but only modestly alters SIV replication kinetics[10]. Although findings in the present study suggest that there is significant variability in the early reverse transcription block between individuals in rhesus monkey populations, there is little evidence of rhesus monkey APOBECs or TRIM5alpha alleles exhibiting a differential ability to block SIV replication *in vitro *nor *in vivo *[14,15]. Our data demonstrate that lymphocytes of rhesus monkeys express an inhibitor of SIV early reverse transcription that is associated with a reduced *in vivo *viral set point, CD4+ T cell decline, and a delay in the time to death following SIV infection. Whether differences in lymphocyte susceptibility to SIV represent consequences of allelic forms or variable expression levels of an SIV restricting molecule, our findings underscore that an innate antiviral response, which is capable of inhibiting early RT, can impact the *in vivo *clinical outcome of the animals infected with SIV. A complete understanding of the host immune mechanisms that have a significant impact on *in vivo *viral replication is critically important to aid in our design and implementation of preventative and therapeutic interventions to reduce HIV acquisition and viral burden.

## Competing interests

The authors declare that they have no competing interests.

## Authors' contributions

TR conceived of and designed the study as well as participated in all assays. SL participated in B-LCL phenotyping and staging assay. TS participated in B-LCL phenotyping, staging, and fusion assay. TC participated in staging and fusion assay. AH participated in *in vivo *correlation studies. All authors have read and approved the manuscript.
